# Role of Procalcitonin for Early Discrimination Between Necrotizing Fasciitis and Cellulitis of the Extremities

**DOI:** 10.7759/cureus.57668

**Published:** 2024-04-05

**Authors:** D Vaibhavi, Sreeramulu P N, Neha Ullalkar, Gurugubelli Amarnath

**Affiliations:** 1 General Surgery, Sri Devaraj Urs Medical College, Kolar, IND; 2 Surgery, R L Jalappa Hospital and Research Centre, Kolar, IND

**Keywords:** necrotizing fasciitis, antibiotics, necrosis, procalcitonin, cellulitis

## Abstract

Introduction

Necrotizing fasciitis (NF) is a grave and life-threatening infection of the soft tissues. It is defined by the gradual necrosis of the fascia and subcutaneous tissue, which spreads along the fascial planes. Cellulitis, a prevalent skin infection, has led to suggestions that procalcitonin could serve as a diagnostic tool to distinguish it from other inflammatory skin conditions that resemble cellulitis. The study aims to assess the procalcitonin (PCT) levels in individuals with NF and cellulitis and determine its effectiveness in early differentiation between these two conditions.

Methods

After obtaining clearance from the institutional ethical committee, the study was conducted in the Department of General Surgery, Sri Devaraj Urs Medical College, over six months. Informed consent was obtained from all 30 patients included in this study. The study compared PCT levels in patients diagnosed with NF and cellulitis. Statistical analysis was performed using SPSS version 22 software (IBM Corp., Armonk, NY, USA).

Results

The mean age of subjects was 53.23 ± 8.78 years. Among patients, 21 (70%) were diagnosed with cellulitis and 9 (30%) were diagnosed with NF. The mean PCT levels were 0.34 ± 0.32 and 4.89 ± 1.98 among the cellulitis and NF groups, respectively. There was a significant difference (p<0.05). PCT had a sensitivity of 100% and a specificity of 100%, in differentiating cellulitis and necrotizing fasciitis.

Conclusion

PCT levels were notably elevated in cases of NF compared to cellulitis. Despite the study's limited sample size, it represents the first report highlighting the value of PCT as an early diagnostic tool for identifying necrotizing fasciitis.

## Introduction

Necrotizing fasciitis (NF) is a grave and life-threatening infection of the soft tissues. It is defined by the gradual necrosis of the fascia and subcutaneous tissue, which spreads along the fascial planes [1]. NF has a significant mortality rate, ranging from 19% to 30% across all NF-affected areas, which encompass the neck, trunk, perineum, and extremities [2-6]. The timely identification of NF is essential for performing a proactive surgical debridement, thereby reducing the patient's mortality risk and the likelihood of amputations. Nevertheless, distinguishing NF from non-necrotizing soft tissue infections (NFTI) can be a challenging task. The gold standard for diagnosing NF relies on a patient's clinical features, surgical examination, as well as a microbiological and histopathological analysis of soft tissue [1]. Efficiently managing NSTI begins with accurately diagnosing the condition. Identifying NSTI can pose challenges, as the initial symptoms, including swelling, redness, and pain, lack specificity and can be associated with various infection types [7,8]. Distinguishing an early diagnosis of NSTI from other soft tissue infections with a more favorable prognosis, such as limb cellulitis, can be a challenging task.

In 2004, a diagnostic scoring system known as the Laboratory Risk Indicators for Necrotizing Fasciitis (LRINEC) score was developed to serve this specific objective [3]. The LRINEC score was developed to establish a straightforward and objective scoring system, utilizing standard laboratory parameters, to differentiate NSTI from other soft tissue infections. Nevertheless, subsequent validity investigations revealed that the LRINEC's accuracy had been exaggerated, with sensitivities ranging from 43.2% to 80.0% for a score of ≥6 and from 28.6% to 68.4% for a score of ≥8 in various settings, countries, or regions [9-11]. Su et al. did not find LRINEC useful for the diagnosis of NF, but they did for its prognosis [12]. Foo et al. reported a low level of diagnosis in immunosuppressed patients [6].

Procalcitonin (PCT) is a peptide that precedes the formation of the hormone calcitonin, which plays a role in maintaining calcium balance in the body. PCT levels rise in response to inflammatory triggers, particularly those caused by bacterial infections [13]. Procalcitonin is a biochemical indicator that becomes elevated in the presence of bacterial infections. While it is primarily generated in thyroidal C-cells and other neuroendocrine cells, it can also be produced by various extra thyroidal cells as a response to bacterial infections [14]. Cellulitis, a prevalent skin infection, has led to suggestions that procalcitonin could serve as a diagnostic tool to distinguish it from other inflammatory skin conditions that resemble cellulitis [15]. Al-Thani et al. reported a positive correlation between LRINEC and PCT levels in predicting septic shock in patients with NF [16,17]. Hence, the study aims to assess the PCT levels in individuals with NF and cellulitis and determine its effectiveness in early differentiation between these two conditions.

## Materials and methods

Study design, sample size, and source of data

This was a prospective study carried out on 30 patients with superficial lower limb infections (NF and cellulitis) in the Department of General Surgery, Sri Devaraj Urs Medical College, over six months (May 2023 to October 2023).

Inclusion and Exclusion Criteria

Table 1 lists the inclusion and exclusion criteria of this study.

**Table 1:** Inclusion and exclusion criteria

Inclusion criteria	Exclusion criteria
Patients with suspected lower limb cellulitis	Patients with deeper infections
Patients with suspected lower limb necrotizing fasciitis	Patients on steroids
Patients who are willing to give written informed consent	Patients who are not willing to give written informed consent

Method of Data Collection

The research followed the guidelines outlined in the 2013 revision of the Helsinki Declaration. The institutional ethical committee, Sri Devaraj Urs Academy of Higher Education and Research, approved the study and granted permission to start of study with approval number DMC/KLR/IEC/184/2023-24 on May 26, 2023. All study participants provided their informed consent.

In accordance with the criteria mentioned earlier, our study included a total of 30 patients of which 9 (30%) had NF and 21 (70%) had cellulitis. We collected comprehensive clinical history. In the case of all enrolled patients, the variables gathered for data analysis were categorized into laboratory values and vital signs observed during their presentation at the emergency department. Data were collected from patients in both groups, encompassing information, such as gender, age, infection site, comorbidities, along with PCT levels upon admission.

Cellulitis was defined in accordance with the criteria established in the Prophylactic Antibiotics for the Treatment of Cellulitis at Home trial (PATCH trial), including the presence of local warmth, tenderness, or acute pain; either unilateral or bilateral erythema, with a clear temporal connection between symptoms and the more affected leg; as well as unilateral edema. In cases where the diagnosis was uncertain, the patient was excluded from the study. Procalcitonin levels were assessed using either the VIDAS® system (bioMérieux, Marcy-l'Étoile, France) or the B.R.A.H.M.S PCT^TM^ system (Roche Diagnostics, Basel, Switzerland). The key focus of the study was to measure procalcitonin levels in all individuals with superficial lower limb infections and then evaluate its effectiveness in distinguishing between lower limb cellulitis and necrotizing fasciitis during the early stages of the conditions. The secondary outcome was to measure the procalcitonin levels before and after the treatment.

Statistical Analysis

Data were entered into a Microsoft Excel data sheet (Microsoft Corporation, Redmond, WA, USA) and were analyzed using SPSS version 22 software (IBM Corp., Armonk, NY, USA). Categorical data were represented in the form of frequencies and proportions. Continuous data were represented as mean and standard deviation. The normality of the continuous data was tested by the Kolmogorov-Smirnov test and the Shapiro-Wilk test. An independent t-test was the test of significance for independent variables. The validity of the screening test was plotted by receiver operator characteristic (ROC) curve sensitivity with specificity, at best, showing the cut-off. Microsoft Excel and Microsoft Word were used to obtain the ROC curve. A p-value (probability that the result is true) of <0.05 was considered statistically significant after assuming all the rules of statistical tests.

## Results

The study comprised 30 patients, and their overall baseline characteristics have been presented in Table 2. The mean age of subjects was 53.23 ± 8.78 years. Fourteen (46.7%) subjects were in the age group of 51 to 60 years. The study included 19 (63.3%) males and 11 (36.7%) females. Twenty-one (70.0%) subjects had cellulitis and 9 (30.0%) had necrotizing fasciitis.

**Table 2:** Baseline characteristics of all participants (n = 30)

Variables		Frequency (n)	Percentage (%)
Age group	<40 years	4	13.3%
	41 to 50 years	7	23.3%
	51 to 60 years	14	46.7%
	>60 years	5	16.7%
Gender	Male	19	63.3%
	Female	11	36.7%
Diagnosis	Cellulitis	21	70.0%
	Necrotizing fasciitis	9	30.0%

The baseline characteristics are further divided into two groups, the NF group and the cellulitis group. The comparison of characteristics is summarized in Table 3. In both groups, the majority were males and belonged to the age group of 51-60 years. There was no statistically significant difference in mean age between the two groups (p > 0.05).

**Table 3:** Comparison of baseline characteristics between two groups

Variables	Necrotizing Fasciitis (9)	Cellulitis (21)	P-value
Age group, n (%)			0.96
<40 years	1 (11.1%)	3 (14.3%)	
41 to 50 years	2 (22.2%)	5 (23.8%)	
51 to 60 years	4 (44.4%)	10 (47.6%)	
>60 years	2 (22.2%)	3 (14.3%)	
Mean age (years)	54.78 ± 7.89	52.57 ± 9.23	0.51
Gender			0.06
Male	8 (88.9%)	11 (52.4%)	
Female	1 (11.1%)	10 (47.6%)	

Procalcitonin values were significantly higher in the necrotizing fasciitis group as compared to the cellulitis group as shown in Table 4.

**Table 4:** Comparison of procalcitonin (µg/L) between two groups * p < 0.001 is statistically significant.

Group	Minimum	Maximum	Mean	SD	p-value
Cellulitis	0.10	1.22	0.34	0.32	< 0.001 *
Necrotizing fasciitis	1.56	7.23	4.89	1.98	

Interpretation

The distribution was normal for the procalcitonin variable; therefore, the student's t-test was applied to compare the two groups. The student t-test was run to determine if there were differences in the procalcitonin level between the NF and cellulitis groups. The mean procalcitonin levels were high in the NF group (4.89 µg/L) compared to the cellulitis group (0.34 µg/L) and were statistically significant (p < 0.001). To explore the relationship between procalcitonin levels and clinical or laboratory measurements, the natural logarithm of procalcitonin values was employed. The magnitude of the correlation coefficient indicates the strength of the relationship between procalcitonin and a specific variable while the p-value quantifies the statistical significance of this observed correlation.

**Figure 1:**
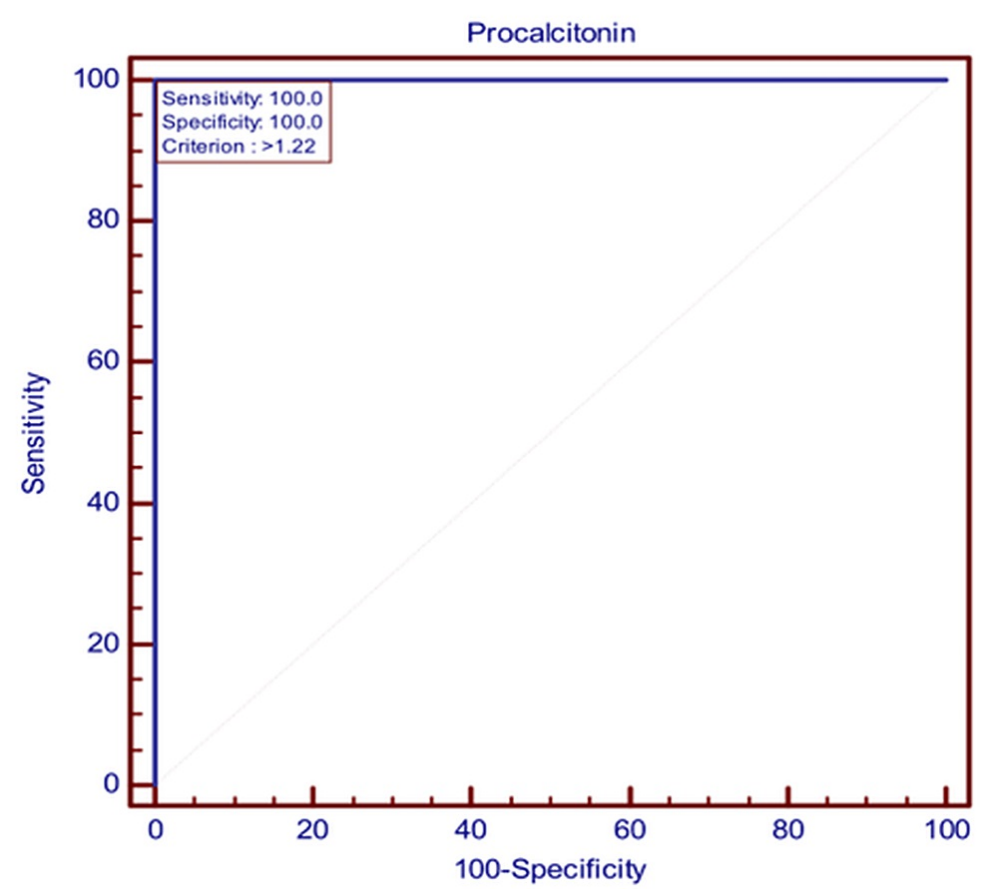
The receiver operator characteristic (ROC) curve showing the area under the curve (AUC) for procalcitonin in differentiating cellulitis and necrotizing fasciitis

ROC curves were generated to assess the sensitivity and specificity of procalcitonin levels. Patients with higher procalcitonin levels were less likely to experience improvement. The ROC curve analysis established a cutoff point of 1.22 for distinguishing between NF and cellulitis. At this threshold, the sensitivity and specificity of procalcitonin (PCT) were both 100%. The positive and negative predictive values (PPV and NPV) were 100% each at this cut-off. At 7.23 PCT as the cut-off point, the sensitivity, specificity, PPV, and NPV were 0%, 100%, nil, and 70%, respectively. Therefore, at the 1.22 cut-off, there was statistical significance (p < 0.0001) with sensitivity and specificity of 100% each. The area under the curve (AUC) was 1.0 exactly at >1.22 associated criterion (Figure 1).

## Discussion

Swift diagnosis and the prompt initiation of surgical debridement are crucial components of the treatment for NSTI in patients facing this severe and potentially life-threatening condition. Identifying NSTI can be particularly challenging when the clinical presentation closely resembles cellulitis [18]. This study explores the role of PCT in early discrimination between necrotizing fasciitis and cellulitis of the extremities.

In this study, the mean age of participants was 53.23 ± 8.78 years. Mean procalcitonin levels were 1.70 ± 2.37 µg/L. Among cellulitis patients, mean procalcitonin levels were 0.34 ± 0.32 µg/L, and among necrotizing fasciitis patients, the mean procalcitonin levels were 4.89 ± 1.98 µg/L. There was a significant difference in mean procalcitonin levels between the two groups. In the research, a procalcitonin level exceeding 1.22 demonstrated a sensitivity, specificity, PPV, and NPV of 100% in distinguishing between cellulitis and necrotizing fasciitis.

Procalcitonin is well-regarded as a valuable biomarker with a significant role in antimicrobial stewardship. We aimed to investigate its relevance in the context of cellulitis. If the procalcitonin test allowed us to confirm the presence of an ongoing bacterial infection, it would enhance the diagnostic accuracy of cellulitis and provide further justification for antibiotic treatment [19].

In another study by Bertolus C et al., there is evidence suggesting that procalcitonin was not deemed a valuable biomarker for risk stratification in cases of facial cellulitis [20]. Only 9% of their patients had procalcitonin values exceeding the clinical threshold of 0.25 µg/L.

In a study conducted by Noh et al., they examined the duration of hospitalization and found a notable correlation with procalcitonin, length of hospital stays, as well as C-reactive protein and total white cell count [21]. Since procalcitonin levels are often associated with the bacterial load, one could anticipate elevated levels in cases of invasive infections. Consequently, this suggests its potential utility in differentiating cellulitis from necrotizing fasciitis.

In a limited-scale study by Kato et al., they determined a cutoff range between 5.88 and 19.96 for procalcitonin levels, which achieved 100% sensitivity, 100% specificity, 100% positive predictive value, and 100% negative predictive value in the differentiation of NF from cellulitis [22]. It is important to note that procalcitonin levels can be elevated due to various factors, including bacterial infections, severe physical trauma, extensive burns, inflammation associated with cytokine storms, and a more extensive series conducted by Na et al. [23], both reported a noteworthy difference in procalcitonin levels. These levels were considerably elevated in patients diagnosed with necrotizing fasciitis as compared to those with cellulitis.

Nakafusa et al. emphasized the significance of serum creatine kinase (CK) levels as a prognostic factor in Vibrio vulnificus infection [24]. In our research, we observed that the CK levels in necrotizing fasciitis cases were notably higher in comparison to cellulitis cases. However, the cutoff point we defined had lower sensitivity than both the LRINEC score and PCT value.

The limitation of this study was the sample size was less compared to other similar studies and only PCT was taken as the single parameter in discrimination between necrotizing fasciitis and cellulitis of the extremities. Other parameters like C- reactive protein, neutrophils, and lymphocytes were not considered, which could have altered ROC curve analysis.

## Conclusions

In summary, our study revealed a substantial elevation in procalcitonin (PCT) levels in cases of necrotizing fasciitis (NF) compared to cellulitis. It explores the role of procalcitonin in early discrimination between NF and cellulitis. Early diagnosis will help in reducing the morbidity and mortality of patients with NF. While our examination involved a relatively small patient population, this represents the initial report highlighting the utility of PCT in the early detection of NF. Further research is warranted to establish an appropriate PCT cutoff value for effectively distinguishing between NF and cellulitis.
